# Complementary Time-Kill Activity Displayed by Povidone-Iodine and Hydrogen Peroxide Against Shoulder Arthroplasty–Associated Pathogens, Envisaging Intraoperative Irrigation

**DOI:** 10.3390/ph19081197

**Published:** 2026-07-30

**Authors:** Enrico Bellato, Filippo Castoldi, Lucrezia Massobrio, Valentina Bartolotti, Carmelo Giannone, Helena Villavicencio, Narcisa Mandras, Alessandro Bondi, Francesca Menotti, Giuliana Banche, Antonio Curtoni, Valeria Allizond

**Affiliations:** 1 Department of Surgical Sciences, University of Torino, 10126 Turin, Italy; enrico.bellato@unito.it (E.B.); filippo.castoldi@unito.it (F.C.); carmelo.giannone@unito.it (C.G.); 2 Department of Public Health and Pediatrics, University of Torino, 10126 Turin, Italy; lucrezia.massobrio@unito.it (L.M.); valentina.bartolotti@unito.it (V.B.); helena.villavicencio@unito.it (H.V.); narcisa.mandras@unito.it (N.M.); alessandro.bondi@unito.it (A.B.); francesca.menotti@unito.it (F.M.); antonio.curtoni@unito.it (A.C.); valeria.allizond@unito.it (V.A.)

**Keywords:** total shoulder arthroplasty, prosthetic joint infection, intraoperative irrigation, povidone–iodine, hydrogen peroxide, time-kill assay, CoNS, *Cutibacterium acnes*

## Abstract

**Background**: Prosthetic joint infection (PJI) following total shoulder arthroplasty (TSA) is a rare but clinically relevant complication, largely attributable to the distinctive shoulder skin microbiome, predominantly composed of *Cutibacterium acnes* and coagulase-negative staphylococci (CoNS), which are considered low-virulence pathogens. Standard skin disinfection protocols do not completely prevent bacterial infiltration into the surgical field; therefore, the World Health Organization (WHO) recommends the use of intraoperative irrigation to reduce the risk of shoulder PJIs. Recently, the use of povidone–iodine (PVI) irrigation has been shown to significantly reduce both bacterial load and diversity after TSA; however, its activity against *C. acnes* remains suboptimal. Therefore, the aim of this study was to evaluate improved in vitro disinfection protocols by combining PVI and hydrogen peroxide for use as intraoperative irrigation in shoulder arthroplasty, which is characterized by a distinctive microbiome. **Methods**: In vitro broth dilution assays and time-kill experiments were performed to test PVI and H_2_O_2_, alone or in combination, against CoNS, *Staphylococcus aureus*, and *Escherichia coli* as representative aerobic bacteria, and against *C. acnes* as an anaerobic pathogen. **Results**: The results revealed that, when used alone, PVI exerted a more pronounced bactericidal effect against staphylococci, whereas H_2_O_2_ was more effective against *C. acnes*, even at a high bacterial inoculum, although at cytotoxic concentrations, particularly for PVI. Notably, when the disinfectants were used in combination, bacterial killing was achieved against all the tested pathogens, starting from short exposure times, mainly 3 min, and at low concentrations. Moreover, the antiseptics significantly reduced mature biofilm, although complete eradication was not achieved. **Conclusions**: A targeted irrigation protocol to prevent shoulder PJI can be achieved by combining PVI and H_2_O_2_ at non-toxic concentrations and for short exposure times that do not interfere with operating room costs or increase the risk of infection.

## 1. Introduction

Despite the implementation of several preoperative strategies and advances in surgical procedures, periprosthetic joint infection (PJI) remains a clinically relevant complication after TSA and is associated with increased healthcare costs, prolonged pain, and impaired function [1,2,3,4,5]. In this context, revision surgery remains the most common approach to treat this complication, despite impacting both patients and healthcare costs [6,7]. The shoulder is characterized by a typical skin microbiome compared with the hip and knee; consequently, PJI in TSA is predominantly due to low-virulence pathogens, mainly *Cutibacterium acnes*, the most commonly isolated bacterium in shoulder arthroplasty [8,9,10,11,12]. *C. acnes* resides in the deep layers of sebaceous glands and hair follicles of the shoulder and is an anaerobic, slow-growing pathogen; these characteristics make its eradication before TSA particularly complex [13,14]. In addition to *C. acnes*, coagulase-negative staphylococci (CoNS), mainly *Staphylococcus epidermidis*, are increasingly implicated in shoulder PJI as facultative anaerobic bacteria. Although less frequently isolated, *Staphylococcus aureus* and *Escherichia coli* have also been identified as causative pathogens of shoulder prosthetic joint infections. The simultaneous presence of anaerobic and aerobic microorganisms in the shoulder surgical field may facilitate their penetration from the incision site into deeper tissues during surgery [3,8,9,15,16].

The distinctive shoulder skin microbiome in TSA, compared with hip and knee surgeries, leads to more challenging preventive strategies [17]. Several strategies alongside standard antimicrobial therapy have been proposed so far to reduce both the microbial load and diversity in these procedures, including preoperative skin disinfection protocols and intraoperative field irrigation [18,19,20,21]. The use of diluted povidone–iodine lavage prior to wound closure in hip and knee arthroplasty is well documented, demonstrating a reduction in both contamination and infection compared with saline alone [22,23,24,25,26].

Building on this evidence, recent research from our group—the first in the field of shoulder arthroplasty—demonstrated that diluted povidone–iodine (PVI) irrigation of the surgical site during TSA significantly reduced both bacterial load and diversity. In particular, it was observed that PVI displayed greater activity against aerobic bacteria, with their load, expressed as colony-forming units (CFU)/mL, being reduced to almost zero. In contrast, *C. acnes* count decreased from 10^3^ to 10^2^ CFU/mL, suggesting lower susceptibility of this anaerobic pathogen to the disinfectant [27]. Moreover, intraoperative irrigation reduced the prevalence of antimicrobial-resistant and biofilm-forming strains of both *C. acnes* and CoNS [28]. These findings support the clinical relevance of this irrigation strategy while highlighting differential responses between aerobic and anaerobic bacteria.

Liang-Gang Li et al. (2024) enrolled patients undergoing total knee arthroplasty and reported that sequential intraoperative irrigation with PVI, hydrogen peroxide (H_2_O_2_), and saline, compared with saline alone, reduces PJI [29]. H_2_O_2_ is a widely used antiseptic solution characterized by potent oxidizing activity and the literature confirms its efficacy against anaerobic microorganisms [30,31]. Since its mode of action differs from that of PVI, providing a complementary activity of the two disinfectants towards aerobic and anaerobic bacteria, the hypothesis that they can be combined for use in irrigation in shoulder arthroplasty—for which the microbiota comprises both pathogen types—has gained greater attention [32,33].

Unfortunately, clinical studies alone are not able to determine the most appropriate disinfectant, concentration, and exposure time to be used in medical practice to achieve optimal antimicrobial efficacy under intraoperative conditions. Similarly, in vitro studies typically determine the minimum inhibitory concentration (MIC) and minimum bactericidal concentration (MBC) after prolonged incubation times—usually around 24 h—which do not reflect the short exposure times characteristic of surgical irrigation during prosthetic implantation, where rapid antimicrobial activity is crucial to prevent infection [30,33]. Moreover, the observed persistence of *C. acnes* after PVI irrigation—despite a reduced bacterial load—confirms the need for combining PVI and H_2_O_2_ to target aerobic and anaerobic bacteria, respectively [27,34,35]. Therefore, assessing their complementary activity under in vitro conditions that reflect clinical settings is crucial to define the most effective evidence-based disinfection protocol for use as intraoperative irrigation immediately after TSA.

The aim of the present study was to investigate the antimicrobial activity of povidone-iodine and hydrogen peroxide—alone and in combination—against shoulder arthroplasty–associated pathogens. Specifically, we sought to (i) characterize the susceptibility of aerobic and anaerobic clinical isolates using MIC and MBC assays, (ii) assess the time-dependent bactericidal activity of each antiseptic—alone and in combination—at clinically relevant concentrations, exposure times, and bacterial inocula, and (iii) identify an optimized time–concentration strategy that could better inform intraoperative irrigation practices in shoulder arthroplasty. As a final aim, we evaluated the most effective combination of PVI and H_2_O_2_ for potentially disrupting mature biofilm of representative clinical strains of *C. acnes* and *S. epidermidis* to evaluate its possible use in cases of PJIs as an alternative to revision surgery.

## 2. Results

### 2.1. MIC and MBC Distribution for Povidone Iodine and Hydrogen Peroxide Against Both Anaerobic and Aerobic Bacteria

At an inoculum of 10^5^ CFU/mL—used as the EUCAST standard condition—PVI showed a MIC_90_ ranging from 0.08% to 0.62%, with slightly higher MBC values (0.62–1.15%) (Table 1). At the lower inoculum (10^3^ CFU/mL), representative of the in vivo shoulder environment during TSA, a more pronounced inhibitory and bactericidal effect was observed (Table 1). Interestingly, H_2_O_2_ exhibited greater inhibitory activity at both inocula, with MBC values (0.003–0.012%) lower than those observed for PVI. For both disinfectants, a clear inoculum-dependent activity was demonstrated (Table 1). Notably, H_2_O_2_ showed both inhibitory and bactericidal effects at lower concentrations compared with PVI, regardless of the inoculum used.

Table 2 and Table 3 report the in vitro activity of both PVI and H_2_O_2_ against CoNS (n = 64). Overall, at 10^5^ CFU/mL, PVI demonstrated the greatest inhibitory effect against *S. epidermidis* (MIC_90_ 0.005–0.313%), whereas the vast majority of CoNS—namely *S. warneri*, *S. capitis*, *S. saprophyticus*, *S. pasteuri*, and *S. lugdunensis*—showed MIC_90_ values ranging from 0.131% to 0.156%; *S. hominis* exhibited MIC_90_ values between 0.01% and 0.313%. At the lower inoculum, a similar pattern was observed, although inhibitory and bactericidal activity was achieved at concentrations one dilution lower. MBC values for PVI were consistently higher than MICs across all CoNS species, ranging from 0.078% to 0.625% (Table 2).

When H_2_O_2_ was assayed against CoNS, a similar inhibitory pattern was observed depending on both the species and the inoculum (Table 3). The most notable results concerned the bactericidal effect, as MBC values were higher than those obtained for PVI, ranging from 0.06% to 2.5% for most CoNS. Accordingly, *S. epidermidis* ATCC 35984 also showed an inoculum-dependent MIC distribution, with values between 0.078 and 0.313% and 0.156–0.313% at 10^3^ and 10^5^ CFU/mL, respectively; in contrast, the MBC was 0.313% at both bacterial concentrations. These data indicate that both PVI and H_2_O_2_ inhibit staphylococcal growth, but higher concentrations of the latter are required to achieve bactericidal activity.

Finally, the activity of PVI and H_2_O_2_ against both clinical and reference strains of *S. aureus* and *E. coli* was determined. Specifically, clinical strains of *S. aureus* and *E. coli* isolated from TSA were assayed together with *S. aureus* ATCC 29213 and *E. coli* ATCC 25922.

For *S. aureus*, no differences were observed between clinical isolates and the ATCC strain for either disinfectant. At 10^5^ CFU/mL, both PVI and H_2_O_2_ showed identical MIC_90_ and MBC values (0.313%), whereas only MIC_90_ decreased to 0.156% at the lower inoculum, indicating an inoculum-dependent effect on inhibitory but not bactericidal activity. A similar behavior was observed for *E. coli*. For both clinical isolates and the ATCC strain, PVI showed MIC values of 0.625% and 0.313% at 10^5^ and 10^3^ CFU/mL, respectively. H_2_O_2_ displayed the same inoculum-dependent trend for MIC, while its MBC decreased from 0.625% at 10^5^ CFU/mL to 0.313% at 10^3^ CFU/mL. In contrast, the bactericidal activity of PVI remained unchanged (0.625%) at both inocula.

### 2.2. Evaluation of the Combination of Povidone-Iodine and Hydrogen Peroxide as Time-Kill Effect Towards C. acnes and CoNS at Different Time Points

When the higher inoculum (10^5^ CFU/mL) was evaluated against *S. epidermidis* and *C. acnes*, a disinfectant concentration- and time-dependent profile was observed. In fact, only PVI at 5% and H_2_O_2_ at 1.5%, used alone or in combination, negatively influenced bacterial growth, with a statistically significant (*p* < 0.001) reduction observed as early as 30 s, at which point no CFUs of either *S. epidermidis* or *C. acnes* were detected. Notably, a similar reduction pattern was also observed when the same bacteria were exposed to high concentrations of PVI and H_2_O_2_, used alone or in combination, at the 10^3^ CFU/mL inoculum. In contrast, at 10^5^ CFU/mL, PVI (0.35% and 0.08%) and H_2_O_2_ (0.5% and 0.012%) used alone failed to exert a bactericidal effect, even after exposure times of up to 10 min. Only the intermediate combination of PVI 0.35% and H_2_O_2_ 0.5% was able to kill both *S. epidermidis* and *C. acnes* after 5 min. A similar concentration- and time-dependent pattern was observed for the other CoNS (*S. warneri*, *S. hominis*, *S. capitis*, *S. saprophyticus*, *S. lugdunensis*, and *S. pasteuri*) included in the analysis, as well as for the *S. epidermidis* ATCC 35984 reference strain.

**Figure 1:**
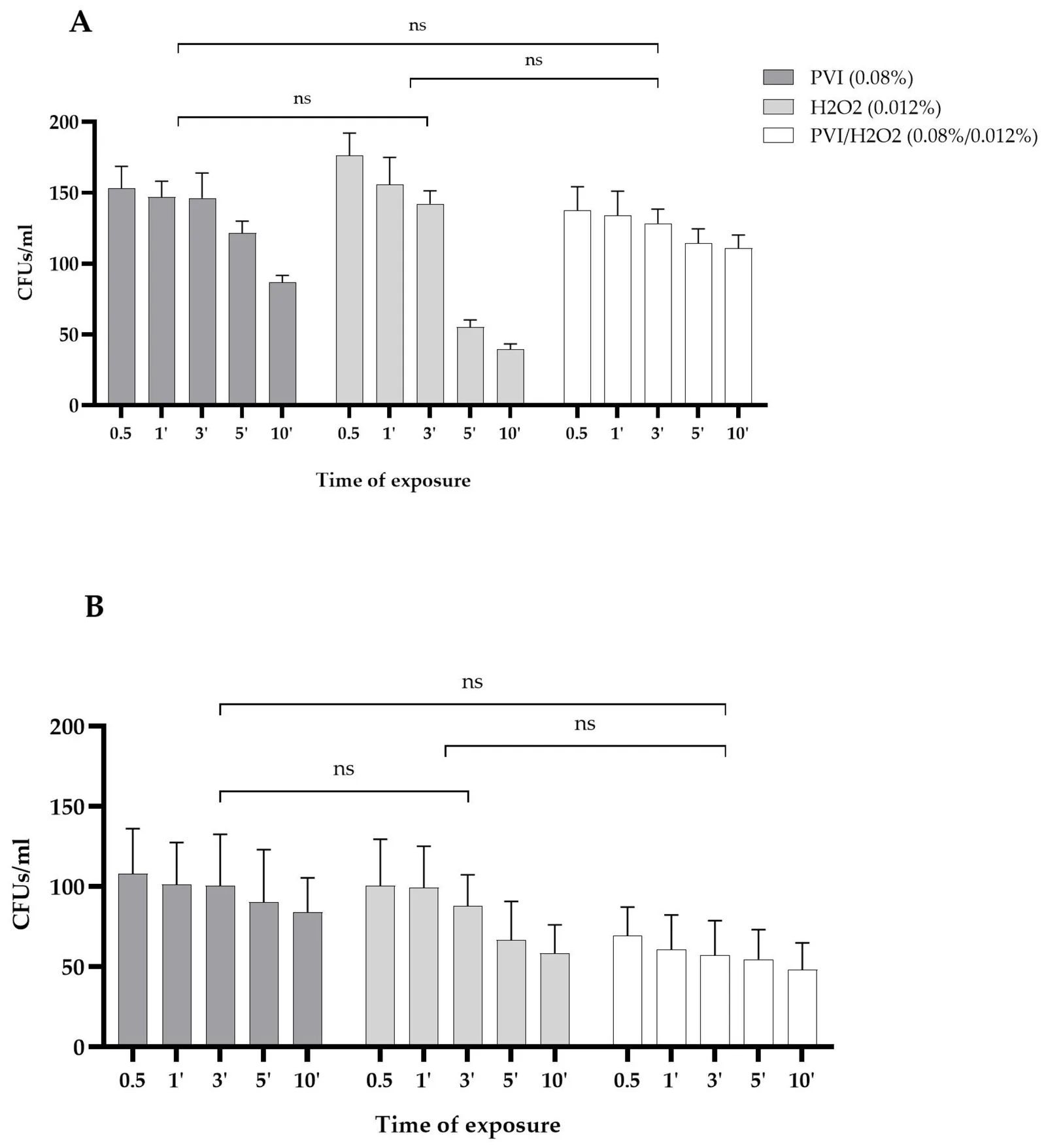
Effect of low disinfectant concentrations, used alone or in combination, on bacterial survival expressed as CFU/mL during time-kill assays (0.5 to 10 min) in *S. epidermidis* (**A**) and *C. acnes* (**B**). ns, not significant.

As depicted in Figure 1, at the 10^3^ CFU/mL inoculum, no statistically significant effect of low concentrations of PVI and H_2_O_2_, used alone or in combination, was observed, as a relevant number of *S. epidermidis* (Figure 1A) and *C. acnes* (Figure 1B) remained detectable throughout the experiment, although a more pronounced reduction was observed for the latter. Comparable results were observed for the remaining CoNS isolates and for the *S. epidermidis* ATCC 35984 reference strain.

**Figure 2:**
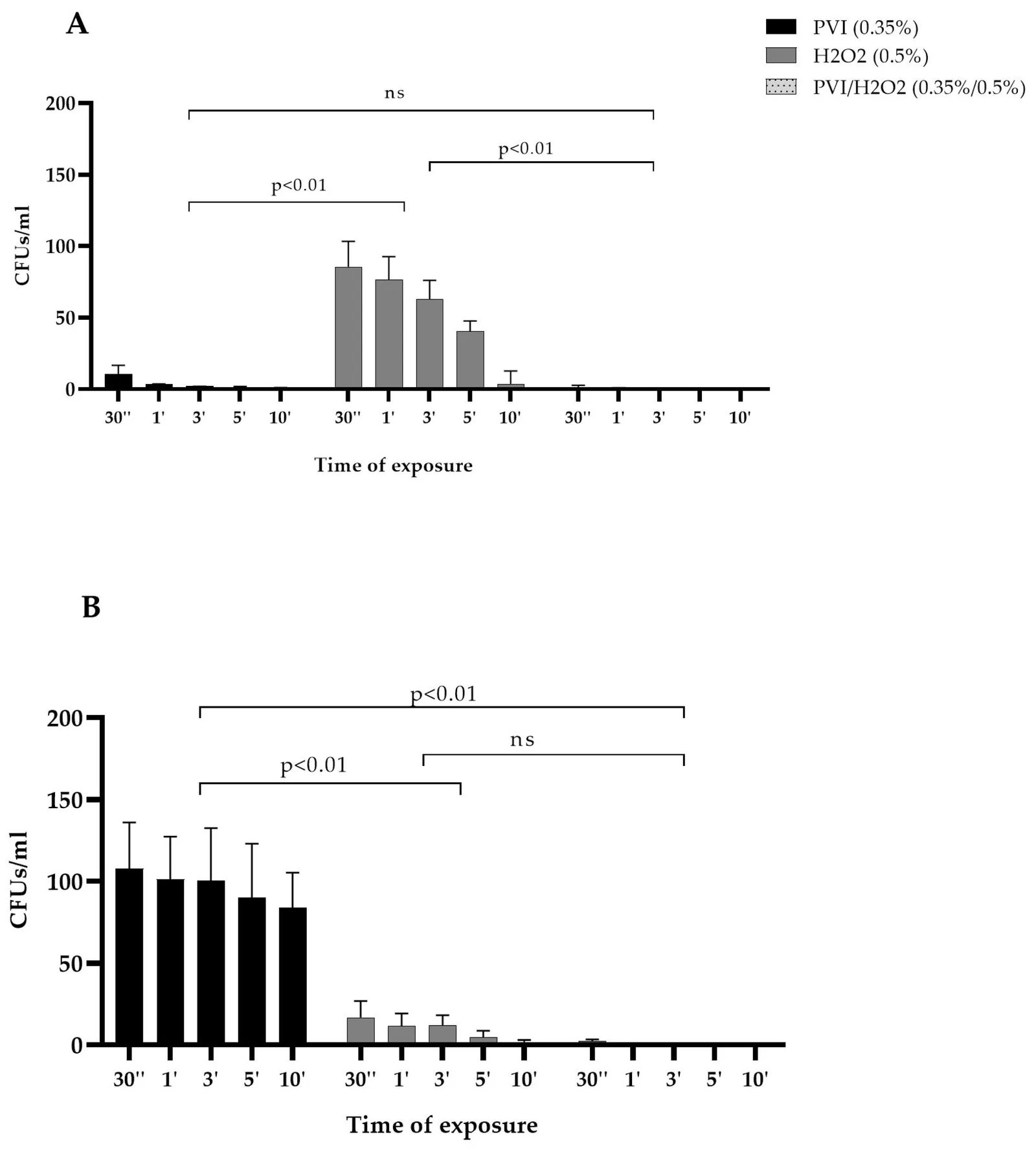
Effect of intermediate disinfectant concentrations, used alone or in combination, on bacterial survival expressed as CFU/mL during time-kill assays (0.5 s to 10 min) in *S. epidermidis* (**A**) and *C. acnes* (**B**). ns, not significant.

**Figure 3:**
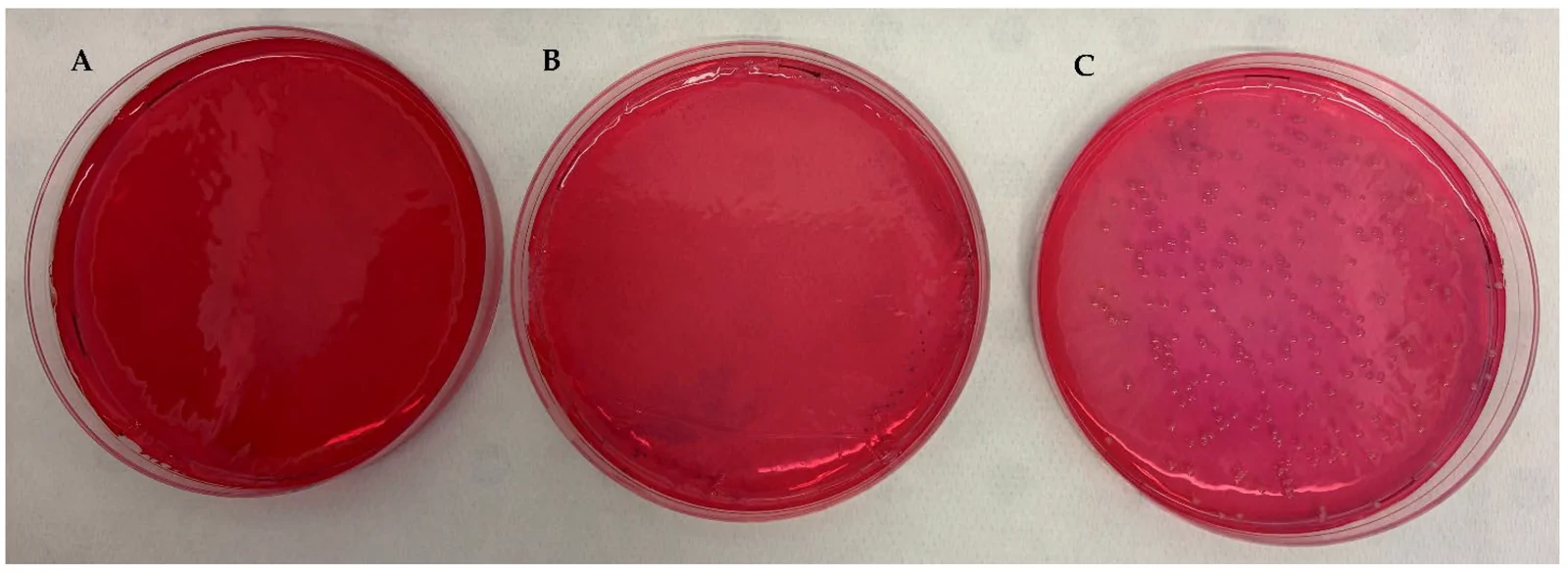
Representative images depicting the time-kill activity of different disinfectant combinations: PVI 5%/H_2_O_2_ 1.5% (**A**), PVI 0.35%/H_2_O_2_ 0.5% (**B**), and PVI 0.08%/H_2_O_2_ 0.012% (**C**), towards *S. epidermdis* growth inhibition after 3 min on mannitol salt agar.

**Figure 4:**
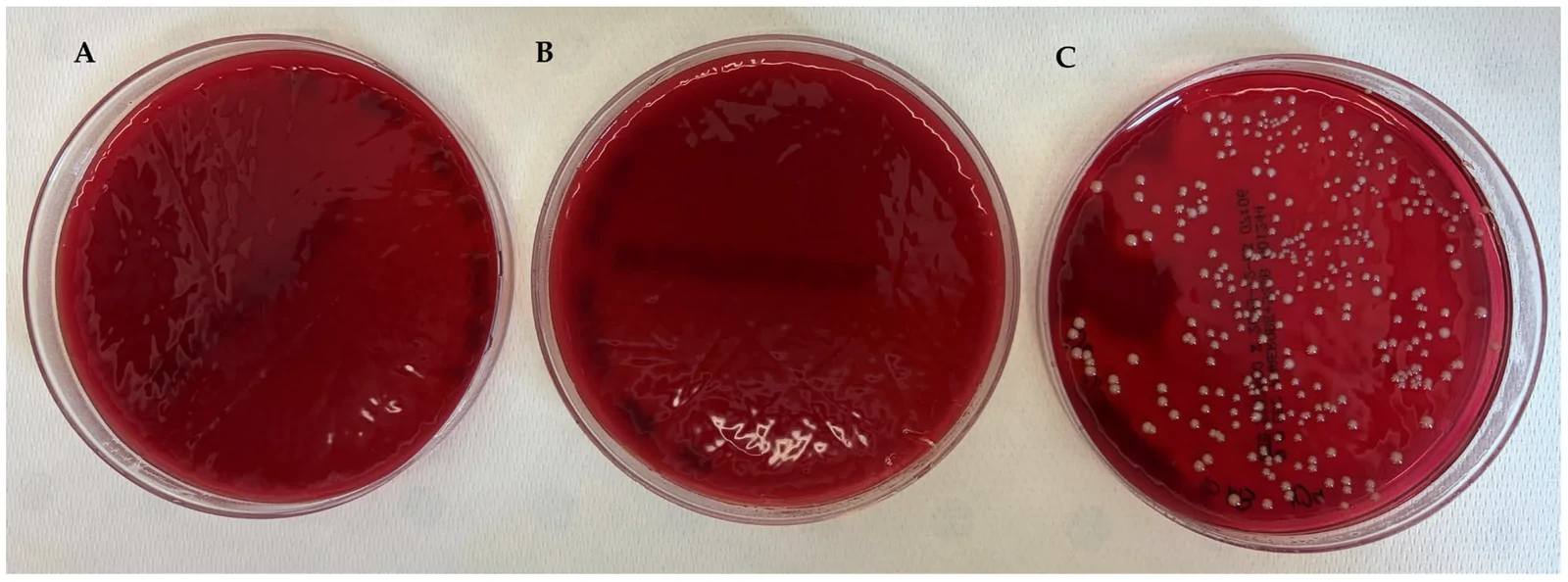
Representative images depicting the time-kill activity of different disinfectant combinations: PVI 5%/H_2_O_2_ 1.5% (**A**), PVI 0.35%/H_2_O_2_ 0.5% (**B**), and PVI 0.08%/H_2_O_2_ 0.012% (**C**), towards *C. acnes* growth inhibition after 3 min on CDC agar.

The most relevant findings emerged at intermediate concentrations (Figure 2). Under these conditions, time-kill assays showed a differential susceptibility between *S. epidermidis* and *C. acnes*, in line with the results observed in the in vitro MBC experiments. Specifically, PVI alone was more effective against *S. epidermidis* (Figure 2A), whereas *C. acnes* (Figure 2B) appeared more sensitive to hydrogen peroxide alone, with a more pronounced bactericidal effect starting from 3 min (Figure 3 and Figure 4). Notably, the combined treatment at intermediate concentrations achieved complete killing of both *S. epidermidis* (*p* < 0.01) and *C. acnes* (*p* < 0.01), with a statistically significant effect observed as early as 0.5 min (Figure 2). As summarized in Table 4 and Table 5, a significant difference (*p* < 0.01) was observed between lower and higher concentrations of PVI against CoNS, H_2_O_2_ against *C. acnes*, and their combination against both bacteria at all incubation times. Comparable results were observed for the remaining CoNS isolates and for the *S. epidermidis* ATCC 35984 reference strain.

**Figure 5:**
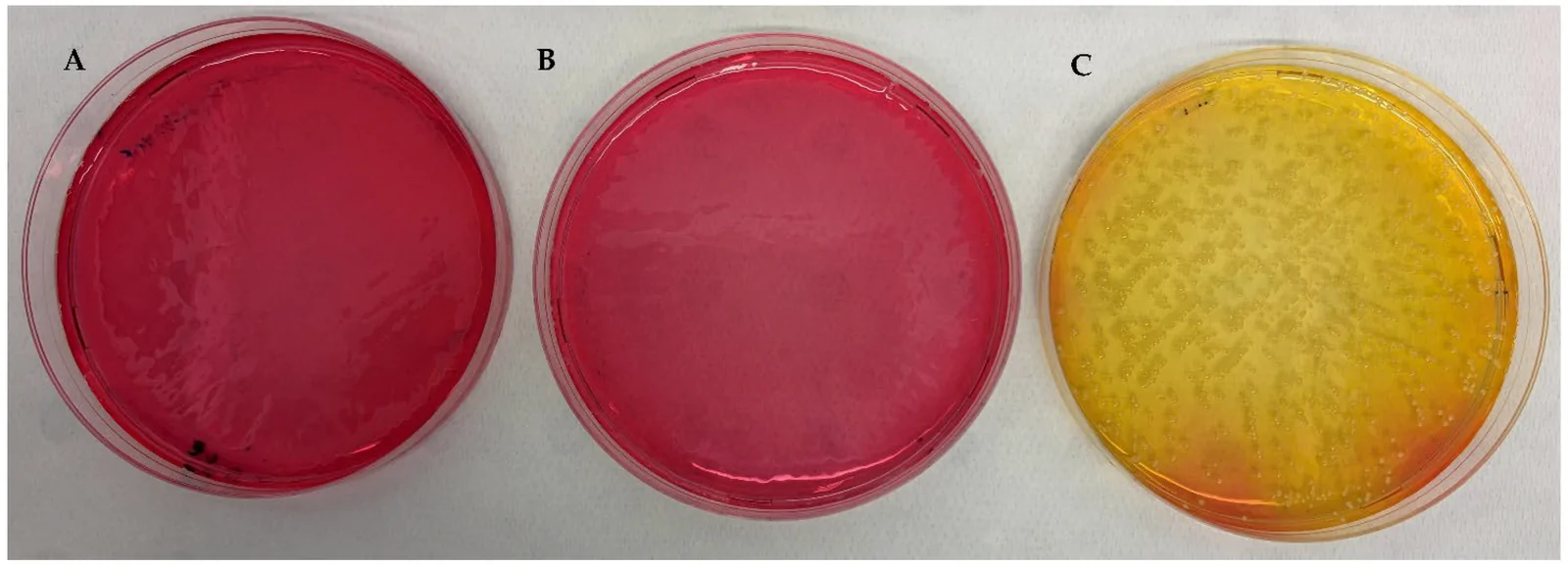
Representative images depicting the time-kill activity of different disinfectant combinations: PVI 5%/H_2_O_2_ 1.5% (**A**), PVI 0.35%/H_2_O_2_ 0.5% (**B**), and PVI 0.08%/H_2_O_2_ 0.012% (**C**), towards *S. aureus* growth inhibition after 3 min on mannitol salt agar.

With regard to *S. aureus*, both the clinical isolate and the *S. aureus* ATCC 29213 strain showed behaviour comparable to that observed for CoNS at both inocula. At the lower inoculum (10^3^ CFU/mL), PVI alone at the intermediate concentration (0.35%) consistently exerted a bactericidal effect across all tested time points, whereas H_2_O_2_ (0.5%) alone showed a weaker and less consistent activity. Notably, the combined PVI–H_2_O_2_ treatment at intermediate concentration resulted in complete killing as early as 30 s, confirming a rapid bactericidal effect under these conditions (Figure 5).

**Figure 6:**
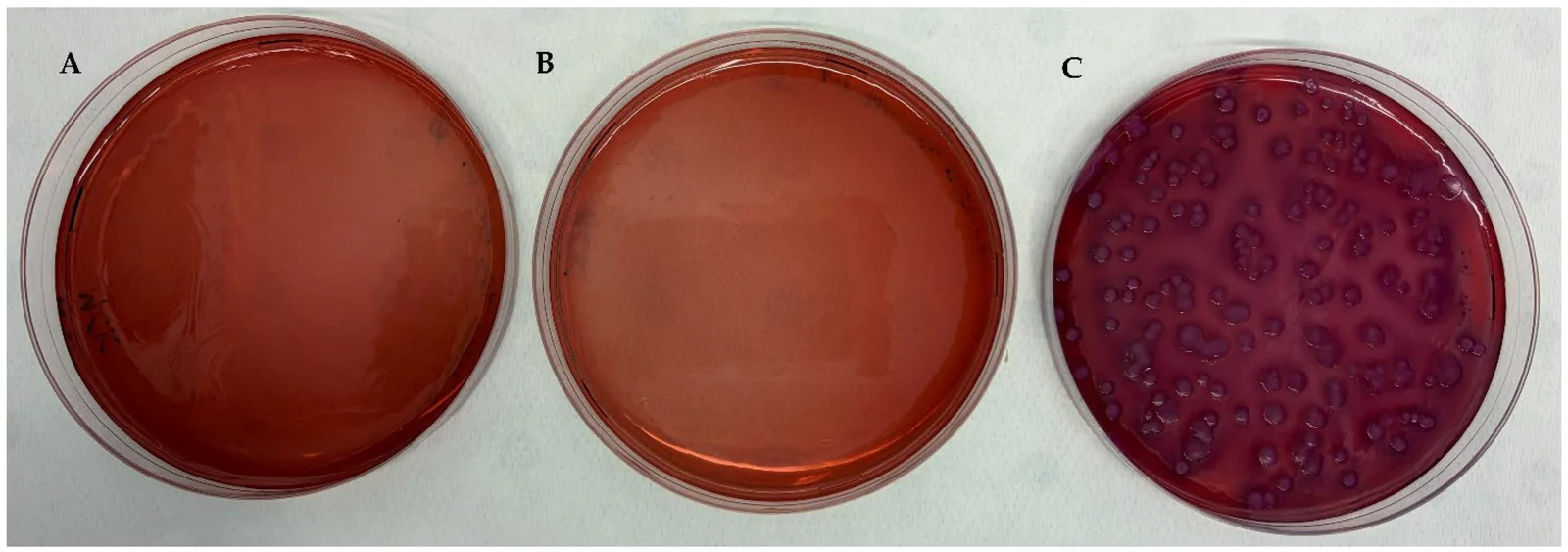
Representative images depicting the time-kill activity of different disinfectant combinations: PVI 5%/H_2_O_2_ 1.5% (**A**), PVI 0.35%/H_2_O_2_ 0.5% (**B**), and PVI 0.08%/H_2_O_2_ 0.012% (**C**), towards *E. coli* growth inhibition after 3 min on MacConkey agar.

On the contrary, a slightly different pattern was observed for *E. coli*. As in MBC assays, *E. coli* showed a somewhat lower susceptibility to PVI and H_2_O_2_ when used individually compared with the other tested organisms. In fact, at the lower inoculum (10^3^ CFU/mL), intermediate concentrations of PVI 0.35% or H_2_O_2_ 0.5% used alone did not induce an immediate bactericidal effect, as viable CFU/mL were still detected at 30 s and 1 min. A progressive reduction in bacterial counts was observed only at longer exposure times, with bactericidal activity becoming evident from 3 min onward (Figure 6). In contrast, the combined PVI 0.35%/H_2_O_2_ 0.5% treatment resulted in complete bacterial killing as early as 30 s, and this effect was maintained across all subsequent exposure times up to 10 min. This behaviour was consistently observed for both the clinical isolate and the *E. coli* ATCC 25922 reference strain.

### 2.3. Correlation Between Susceptibility to Antiseptic Solutions, Antimicrobial Resistance, and Biofilm Formation

In *C. acnes*, no evident correlation was observed between antibiotic resistance patterns and MIC_90_ or MBC values for either povidone-iodine or hydrogen peroxide. Isolates classified as resistant to commonly used antibiotics did not require higher concentrations of either disinfectant to achieve growth inhibition or bactericidal activity. Similarly, biofilm production did not appear to influence antiseptic susceptibility. Isolates with weak or moderate biofilm-forming capacity exhibited MIC and MBC values comparable to those of isolates with lower biofilm production. This lack of association was observed for both disinfectants and at both inocula. Moreover, the analysis of CoNS revealed no consistent association between antibiotic resistance profiles and susceptibility to either povidone-iodine or hydrogen peroxide. Across all CoNS species, isolates exhibiting resistance to one or more conventional antibiotics showed MIC90 and MBC values comparable to those observed in antibiotic-susceptible isolates, at both tested inocula (10^5^ and 10^3^ CFU/mL). Similarly, biofilm-forming capacity did not correlate with reduced susceptibility to the tested antiseptics. Weak and moderate biofilm-producing isolates displayed MIC and MBC values within the same concentration ranges as isolates with lower or negligible biofilm production. This observation was consistent for both povidone-iodine and hydrogen peroxide and across inoculum sizes.

### 2.4. Effect of Antiseptic Solutions on Mature Biofilm at Different Concentrations and Times of Exposure Towards C. acnes and CoNS

**Figure 7:**
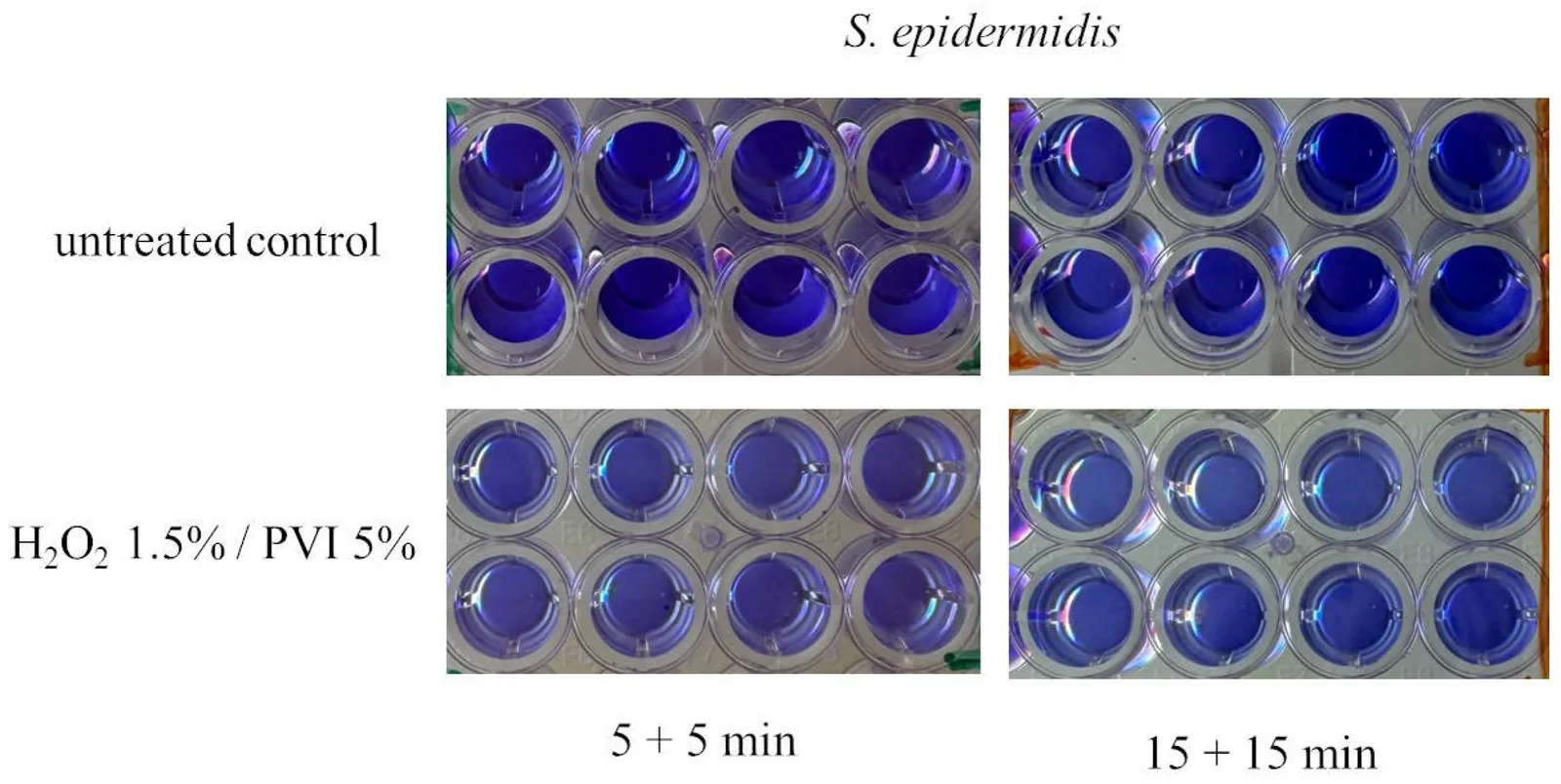
Representative images depicting crystal violet staining of *S. epidermidis* biofilm reduction after exposure, at different time points, to H_2_O_2_ 1.5% followed by PVI 5% (H_2_O_2_ 1.5%/PVI 5%).

**Figure 8:**
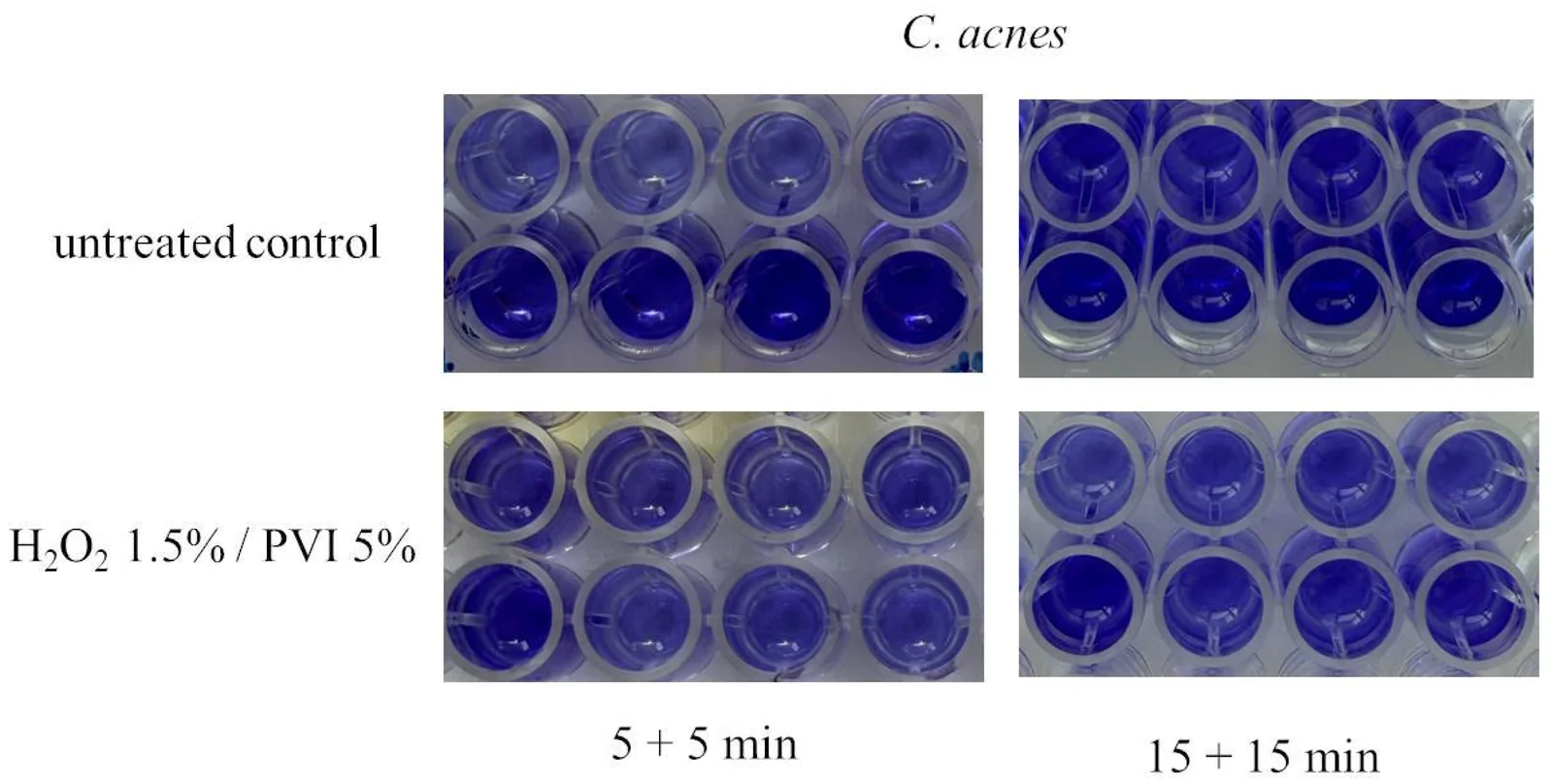
Representative images depicting crystal violet staining of *C. acnes* biofilm reduction after exposure, at different time points, to H_2_O_2_ 1.5% followed by PVI 5% (H_2_O_2_ 1.5%/PVI 5%).

As detailed in Table 6, none of the antiseptic solutions completely eradicated the mature biofilm of either *S. epidermidis* or *C. acnes*. In particular, when the *S. epidermidis* biofilm was treated with the antiseptics, the obtained percentages of reduction ranged from 33% to 44% after 5 min and from 44% to 52% after 15 min when exposed to the lower concentrations; a slightly higher effect was noted when H_2_O_2_ was used before PVI (Figure 7). As expected, higher antiseptic concentrations resulted in greater biofilm reductions, reaching approximately 60% (Table 6 and Figure 7). A similar reduction pattern was observed for *C. acnes*, with biofilm reductions of approximately 40–50%. Also in this case, the highest reduction percentages were achieved when H_2_O_2_ was applied before PVI, particularly after 15 min of exposure (Table 6 and Figure 8).

## 3. Discussion

During TSA, bacteria from the skin can access deeper tissues, leading to PJI, mainly caused by low-virulence organisms that characterize the shoulder skin microbiome, such as *C. acnes* and CoNS, despite standard skin preparation protocols used as preventive strategies [32,36,37]. Moreover, infection can be exacerbated by the ability of these pathogens to produce biofilm on the prosthesis, which protects them from the host immune response and significantly reduces susceptibility to antimicrobials, as well as by the presence of resistant microorganisms [38,39,40]. Literature data suggest the use of intraoperative irrigation to reduce microbial load and diversity in the surgical field immediately after implantation; however, most studies have focused on pathogens commonly isolated after hip and knee arthroplasty, whereas shoulder arthroplasty is also characterized by the presence of anaerobic bacteria [41].

In previous research, we evaluated the impact of PVI irrigation after TSA on bacterial load and diversity, highlighting that CoNS were markedly reduced, whereas *C. acnes*, although significantly decreased, persisted in the operative field [27]. These findings are consistent with studies performed in hip and knee arthroplasty, demonstrating that PVI irrigation effectively reduces bacterial contamination and infection rates, although mainly targeting aerobic pathogens [22,23,26]. Further supporting the role of intraoperative irrigation as a key intervention to reduce bacterial contamination.

In this context, although several in vitro studies have been conducted [38,39,42,43], the optimal irrigation strategy has yet to be defined, particularly in terms of the most effective combination of antiseptics, their concentration, and the exposure time to be applied in clinical practice. Therefore, the present research aimed to evaluate the most promising association of PVI and H_2_O_2_, as well as the effective time of exposure towards bacteria involved in PJIs, particularly those recovered in shoulder arthroplasty.

From our results, both PVI and H_2_O_2_ exhibited bactericidal activity against anaerobic and aerobic bacteria, although with a marked species-dependent pattern: PVI was more effective against staphylococci, whereas H_2_O_2_ showed greater activity against *C. acnes*. Higher concentrations were required for PVI to achieve bactericidal activity against *C. acnes* and for H_2_O_2_ against aerobic bacteria. Regarding *E. coli*, a lower susceptibility to both PVI and H_2_O_2_ was observed. These data are consistent with those of Roscetto et al., who demonstrated that the two disinfectants act differently depending on the bacterial species. In particular, PVI at 5% showed greater activity against Gram-positive bacteria, such as *S. aureus* and *S. epidermidis*, compared with Gram-negative bacteria, such as *Pseudomonas aeruginosa*, whereas H_2_O_2_ exhibited a less consistent activity pattern across bacterial species [38]. Additionally, other studies have highlighted the effectiveness of PVI against biofilm-producing pathogens, including *C. acnes*, although higher concentrations (5–10%) were generally required to achieve a bactericidal effect [44]. These findings demonstrate the efficacy of PVI; however, the concentrations tested are not compatible with clinical practice, since a cytotoxic effect on human cells has already been noted at 5–10% [41,43]. Therefore, a challenge is not only to identify non-toxic concentrations, but also to effectively target different types and loads of pathogens.

A large body of literature has evaluated the microbicidal activity of H_2_O_2_, which acts via a different mechanism than PVI, relying on the release of reactive oxygen species (ROS) that interact with bacterial structures and enzymes, impairing their function [31,38]. In particular, when H_2_O_2_—at 1% or 0.5%—was tested against staphylococci and *P. aeruginosa*, a reduced effect was observed compared with PVI, and its antimicrobial activity was strongly dependent on both exposure time and concentration [38]. Similarly, Menschner et al. demonstrated that ROS derived from H_2_O_2_ are able to exert bactericidal activity against different Gram-positive species when tested at concentrations of 1–3%, with a more pronounced effect on *S. aureus* compared with *Streptococcus pyogenes* [45].

These findings are in agreement with our results, in which H_2_O_2_ showed antimicrobial activity against both CoNS and *S. aureus* compared with *E. coli*. Notably, in our study, H_2_O_2_ exhibited the greatest effect against anaerobic bacteria, particularly *C. acnes*, which was not included in the aforementioned studies [38,45].

Notably, the rationale for the combined use of PVI and H_2_O_2_ lies in their complementary mechanisms of action on bacterial cells; although both agents act through oxidative pathways, they target different microbial structures and, therefore, when used together, enhance their bactericidal effectiveness against Gram-positive and Gram-negative bacteria, together with anaerobes [38,43]. On this basis, the step forward was to perform time-kill experiments to evaluate the most effective combination of PVI and H_2_O_2_ against the common pathogens involved in shoulder PJIs, as well as the optimal exposure time. The results indicated that the combination of PVI (5%) and H_2_O_2_ (1.5%) was able to achieve complete killing of both aerobic and anaerobic bacteria at higher concentrations, with this effect observed as early as 30 s. Moreover, a similar outcome was obtained at intermediate concentrations (PVI 0.35% and H_2_O_2_ 0.5%), although with a slight delay, with 3 min representing the optimal exposure time. Conversely, lower concentrations that were effective when used alone failed to exert a bactericidal effect when combined, except after 10 min of exposure, which exceeds the duration typically feasible in an intraoperative setting. Remarkably, these results were consistent across the different bacterial species tested, further supporting the translational relevance of PVI/H_2_O_2_ against the most common pathogens involved in PJIs of the hip and knee, as well as those associated with the shoulder, particularly *C. acnes*, which resides in the deep layers of sebaceous glands, thus supporting its broad-spectrum activity. In accordance with these observations, other studies have demonstrated a synergistic bactericidal effect of PVI and H_2_O_2_ against both Gram-positive and Gram-negative bacteria, particularly at longer exposure times [38]. However, this study did not take into consideration *C. acnes*, which is a major component of the shoulder skin microbiome.

Finally, the impact of the combination of PVI and H_2_O_2_ on biofilm-forming capability and antimicrobial resistance was investigated: our data showed that exposure of these high-virulence pathogens to both disinfectants did not impair their bactericidal efficacy and did not require prolonged exposure times. These results agree well with Boonchaya et al., who underlined a similar outcome by using an antiseptic solution based on Benzoyl Peroxide [42].

In shoulder arthroplasty, it is very important not only to identify a strategy to prevent PJIs, but also to evaluate whether an irrigation would be able to eradicate the biofilm in case of infection, where revision surgery still represents the only therapeutic option [6,7]. Therefore, we assessed the effect of the antiseptic solutions in eliminating mature biofilm: we demonstrated that the mature biofilm of both aerobic and anaerobic bacteria was not completely eradicated when subjected to PVI and H_2_O_2_ exposure, either at higher concentrations or after prolonged treatment (15 min). In both cases, we noted that H_2_O_2_ was more effective when used before PVI. These data agree well with those of other authors, who reported a reduction, but not a complete eradication, of biofilm, although *C. acnes* was not assayed in all those studies [32,38,39,44].

By hypothesizing the use of PVI and H_2_O_2_ together intraoperatively in the arthroplasty clinical setting, the appropriate balance between killing activity against pathogens and the absence of cytotoxic effects on eukaryotic cells—which may subsequently prolong the healing time—is to be clearly considered. Furthermore, it should be noted that prolonging the duration of irrigation in the operating room increases both the risk of infection and the costs for the national health system. To reinforce the potential translational applicability of this irrigation strategy, the intraoperative use of the combination of PVI and H_2_O_2_ is currently being assessed in patients undergoing TSA. Preliminary results seem to confirm that no local adverse effects or prolonged wound healing have occurred. However, our results do not support the use of the antiseptic solution as an irrigation protocol in cases of infected prostheses instead of implant removal, since the biofilm plausibly present during PJIs was not completely eradicated.

## 4. Materials and Methods

### 4.1. Bacterial Strains and Antiseptic Solutions

The in vitro experiments were performed using both clinical and reference bacterial strains. Clinical isolates were collected from patients undergoing TSA, including both aerobic/facultative anaerobic and anaerobic bacteria. Aerobic clinical isolates comprised coagulase-negative staphylococci (CoNS, n = 64), including *S. epidermidis* (n = 22), *S. warneri* (n = 14), *S. hominis* (n = 12), *S. capitis* (n = 9), *S. saprophyticus* (n = 3), *S. lugdunensis* (n = 2), and *S. pasteuri* (n = 2), together with one *S. aureus* isolate and one *E. coli* isolate. In addition, 120 *C. acnes* clinical isolates were included in the study. All bacterial isolates were identified at the species level using matrix-assisted laser desorption/ionization time-of-flight mass spectrometry (MALDI-TOF MS; Bruker Daltonics, Bremen, Germany). They were also previously characterized to determine antimicrobial susceptibility patterns and biofilm production capability [28]. Minimum inhibitory concentration (MIC) and minimum bactericidal concentration (MBC) determinations were performed using the entire collection of clinical and reference strains. Time-kill assays were performed on a representative subset of the strain collection, selected according to the relative prevalence of the different bacterial species and clinically relevant characteristics, including antimicrobial resistance and/or biofilm-producing ability. As reference strains, *S. epidermidis* ATCC 35984, *S. aureus* ATCC 29213, and *E. coli* ATCC 25922 were included to ensure comparability with other studies. No reference strains were used for *C. acnes*, as available strains were considered not representative of the orthopaedic clinical setting.

Povidone–iodine (PVI 10%, Mylan, Canonsburg, PA, USA) and hydrogen peroxide (H_2_O_2_ 1.5%, Liofilchem, Roseto degli Abruzzi, Italy) were used as test antiseptics. All experiments were performed starting from the commercial stock solutions, prepared according to the manufacturer’s instructions.

### 4.2. Evaluation of Povidone-Iodine and Hydrogen Peroxide Minimum Inhibitory Concentration and Minimum Bactericidal Concentration Towards Bacterial Strains

A custom broth microdilution assay was used to evaluate the antimicrobial activity of PVI and H_2_O_2_, following the principles of the European Committee on Antimicrobial Susceptibility Testing (EUCAST) and adapted for the evaluation of disinfectants. EUCAST definitive document E.Def 7.3.2, 2020. Available online: https://www.eucast.org (accessed on 6 September 2025). For aerobic bacteria (CoNS, *S. aureus* and *E. coli*), susceptibility testing was performed using cation-adjusted Mueller–Hinton broth (CAMHB) (Becton Dickinson, BD, Franklin Lakes, NJ, USA), in accordance with EUCAST recommendations. For *C. acnes*, susceptibility testing was performed using Thioglycollate broth (TG-broth, BD, USA) to support anaerobic growth. The selected media are consistent with standard microbiological practice and previous in vitro studies evaluating antiseptic susceptibility [28,37,42].

Bacterial suspensions were prepared from fresh overnight cultures. Colonies were resuspended in sterile 0.9% NaCl solution and adjusted to the appropriate turbidity. Two different final inocula were used: 10^5^ CFU/mL, corresponding to the standard inoculum recommended by EUCAST for broth microdilution susceptibility testing, and 10^3^ CFU/mL, corresponding to the bacterial concentration detected in vivo in patients undergoing TSA [27]. The use of both inocula allowed comparison between standard in vitro susceptibility conditions and bacterial loads representative of the clinical setting. Inoculum concentrations were confirmed by viable colony counts.

MICs were determined by adapting the broth microdilution method in sterile 96-well microtiter plates for disinfectants [30,33]. Serial dilutions (*v*/*v*) of freshly prepared PVI and H_2_O_2_ were obtained in the appropriate culture media, ranging from 5% to 0.0025% for PVI and from 1.5% to 0.0015% for H_2_O_2_. A volume of 100 µL of disinfectant was mixed with an equal volume of bacterial inoculum. All plates were incubated at 37 °C for 18–24 h for aerobic bacteria, whereas a prolonged incubation time, achieving 48–72 h, was used for *C. acnes*. MIC determination was performed by spectrophotometric reading using a microtiter plate reader (VICTOR™, PerkinElmer, Boston, MA, USA) at a wavelength of 595 nm. The MIC was defined as the lowest concentration of antiseptic that completely inhibited visible bacterial growth. MIC_50_ and MIC_90_ values were calculated as the concentrations inhibiting 50% and 90% of isolates, respectively. In parallel, MBCs were determined from wells showing no visible growth in the MIC assay. A total volume of 200 μL from each selected well was transferred into a neutralizing solution composed of 0.1 g tryptone (Oxoid, Basingstoke, UK), 0.85 g NaCl (Sigma-Aldrich, St. Louis, MO, USA), 0.5 mL Tween 80 (Sigma-Aldrich, St. Louis, MO, USA) and sterile distilled water up to 100 mL, prepared according to Molinar et al. [46], to inactivate residual disinfectant activity. After neutralization, 100 μL of the resulting suspension was plated onto antiseptic-free tryptic soy agar (TSA; Oxoid, Basingstoke, UK) for aerobic bacteria and antiseptic-free TG-agar (BD, Franklin Lakes, NJ, USA) for *C. acnes*, followed by incubation under the appropriate atmospheric conditions. The MBC was defined as the lowest concentration of antiseptic resulting in the absence of colony growth on subculture, corresponding to a ≥99.9% reduction in the initial inoculum. All experiments were performed in duplicate and repeated at least three times.

### 4.3. Time-Kill of Different Povidone-Iodine and Hydrogen Peroxide Combinations Towards Clinical and Reference Strains

Time-kill assays were performed to assess the bactericidal activity of PVI and H_2_O_2_, tested alone or in combination, at two bacterial inocula (10^5^ and 10^3^ CFU/mL) for increasing incubation times, from 0.5 to 10 min [38]. Briefly, bacterial inocula were obtained by appropriately diluting fresh cultures to achieve final concentrations of approximately 10^5^ and 10^3^ CFU/mL, and 100 µL were mixed (1:1 ratio) with the disinfectants tested at three different concentrations: high (PVI 5% and H_2_O_2_ 1.5%), intermediate (PVI 0.35% and H_2_O_2_ 0.5%), and low (PVI 0.08% and H_2_O_2_ 0.012%). These concentrations were selected based on MBC experiments, in which PVI and H_2_O_2_ achieved bactericidal activity against CoNS and *C. acnes*, respectively. PVI and H_2_O_2_ were also assayed alone at the same concentrations; additionally, 0.9% saline solution was used as a control condition. At each time point, disinfectant activity was stopped by adding 200 µL of neutralizing solution and allowing it to act for 5 min. Subsequently, 100 µL were plated on Mannitol Salt Agar (MSA; Oxoid, UK) for CoNS and *S. aureus*, and on MacConkey agar (Oxoid, UK) for *E. coli*, and incubated aerobically at 37 °C for 18–24 h; whereas *C. acnes* was plated onto CDC agar (BD, Franklin Lakes, NJ, USA) and incubated under strictly anaerobic conditions using a GasPak system (BD, Franklin Lakes, NJ, USA) for 72 h. After incubation, bacterial load was quantified by counting CFU/mL, and when no colonies were detected, corresponding to the absence of detectable viable bacteria.

Since all strains were previously characterized for their susceptibility to antimicrobial agents as well as their biofilm production capability [28], the final step of the study was to assess the association between these virulence features and susceptibility to the disinfectants.

### 4.4. Effect of the Antiseptic Solution in the Eradication of Mature Biofilm

The experiments were performed to evaluate whether the two antiseptics in combination were able to disrupt either *C. acnes* or *S. epidermidis* mature biofilm. Briefly, fresh overnight bacterial cultures were centrifuged and resuspended in broth to reach a final concentration of about 10^6^ CFU/mL; TG broth (BD, Franklin Lakes, NJ, USA) and BHI (Oxoid, UK) supplemented with 2% glucose were used for *C. acnes* and CoNS, respectively. A 3-day mature biofilm was used for *C. acnes*, whereas a 24 h mature biofilm was obtained for *S. epidermidis*, as previously described in detail [28]. Preliminary experiments were performed by treating the biofilm for 3, 5, 15, and 30 min with the most efficient combination of the two disinfectants, namely PVI 0.35% and H_2_O_2_ 0.5%. Since this combination did not act on the biofilm, another approach was tested. Specifically, the mature biofilm was treated with a sequential exposure to PVI at 5% or 0.35% and then H_2_O_2_ at 1.5% or 0.5%, and vice versa, for 5 + 5 min or 15 + 15 min. After each antiseptic exposure, 200 µL of a neutralizing solution was applied, and three washing steps with 200 µL of sterile water were performed. Thereafter, fixation with methanol was performed, followed by staining with crystal violet (1%) and dissolution with acetic acid (30%). Finally, the plates were read at 595 nm using a VICTOR3™ microplate reader (PerkinElmer, Waltham, MA, USA) to obtain the optical density (O.D.) of each well [28]. In parallel, biofilm untreated with the disinfectants was prepared and used as a control.

The biofilm reduction in disinfectant-treated samples compared with untreated controls was calculated using the following formula: % of reduction = [(Mean O.D. untreated control − Mean O.D. treated sample)/Mean O.D. untreated control] × 100.

### 4.5. Statistical Analysis

MIC and MBC results were reported as ranges of concentrations—reported as *v*/*v* percentages—inhibiting or killing the tested isolates. No inferential statistical analysis was applied to MIC/MBC data, as these assays were intended to provide a descriptive comparison of antimicrobial susceptibility across disinfectants and bacterial species.

For time-kill experiments, bacterial survival was expressed as CFU/mL, and results were summarized as mean ± SEM. The biofilm experiments were reported as mean O.D. values, and the percentage of reduction compared with untreated controls was calculated. Statistical comparisons were performed to evaluate the effect of PVI or H_2_O_2_ used alone or in combination using GraphPad Prism 9.0 (GraphPad Software, San Diego, CA, USA). Comparisons between treatments were carried out using an unpaired *t*-test, as independent samples were analyzed at each exposure time. All statistical analyses were performed using standard statistical software. A *p*-value < 0.05 was considered statistically significant.

## 5. Conclusions

In conclusion, the present research demonstrated that PVI and H_2_O_2_ targeted different pathogens involved in shoulder PJIs, as PVI impaired the viability of aerobic pathogens but did not affect *C. acnes* survival, which was achieved only at concentrations toxic in human clinical applications. On the contrary, H_2_O_2_ displayed a complementary effect by acting effectively against anaerobic bacteria. Notably, the association of these disinfectants at concentrations suitable for the surgical environment and applied for only 3 min highlighted a complete bactericidal effect towards Gram-positive, Gram-negative, and anaerobic bacteria without being impaired by their virulence features, specifically biofilm-forming capability and resistance to antimicrobial agents. These findings provide the basis for the use of this irrigation strategy in future clinical studies during shoulder replacement surgery to significantly reduce the occurrence of PJIs. Nevertheless, this approach is not applicable when PJIs are already ongoing, since it is not able to fully eradicate the biofilm, and revision surgery remains the only possible way to counteract these infections.

## Figures and Tables

**Table 1:** MIC_50_, MIC_90_ and MBC—reported as *v*/*v* percentages—values of povidone-iodine or hydrogen peroxide against *C. acnes* (n = 120) at two different inocula (10^5^ and 10^3^ CFU/mL).

Antiseptic	Inoculum (CFUs/mL)	MIC_50_ Range (% *v*/*v*)	MIC_90_ Range (% *v*/*v*)	MBC Range (% *v*/*v*)
Povidone-iodine	10^5^	0.04–0.31	0.08–0.62	0.62–1.25
	10^3^	0.02–0.04	0.04–0.08	0.156–0.62
Hydrogen peroxide	10^5^	0.0015–0.006	0.003–0.006	0.006–0.012
	10^3^	0.00075–0.003	0.0015–0.003	0.003–0.012

**Table 2:** MIC_50_, MIC_90_ and MBC—reported as *v*/*v* percentages—values of povidone-iodine against coagulase-negative staphylococci (CoNS; n = 64) at two different inocula (10^5^ and 10^3^ CFUs/mL).

CoNS (n = 64)	Inoculum (CFUs/mL)	MIC_50_ Range (% *v*/*v*)	MIC_90_ Range (% *v*/*v*)	MBC Range (% *v*/*v*)
*S. epidermidis* (n = 22)	10^5^	0.0025–0.313	0.005–0.313	0.078–0.625
	10^3^	0.0025–0.156	0.005–0.156	0.156–0.625
*S. warneri* (n = 14)	10^5^	0.078–0.156	0.156–0.313	0.313–0.625
	10^3^	0.078–0.156	0.156–0.313	0.313–1.25
*S. hominis* (n = 12)	10^5^	0.005–0.156	0.01–0.313	0.156–0.625
	10^3^	0.01–0.156	0.039–0.156	0.078–0.625
*S. capitis* (n = 9)	10^5^	0.078–0.156	0.156–0.313	0.313–0.625
	10^3^	0.078–0.156	0.156–0.313	0.156–0.313
*S. saprophyticus* (n = 3)	10^5^	0.078–0.156	0.156–0.313	0.625
	10^3^	0.156	0.313	0.625
*S. pasteuri* (n = 2)	10^5^	0.078–0.156	0.156–0.313	0.625
	10^3^	0.156	0.313	0.313–0.625
*S. lugdunensis* (n = 2)	10^5^	0.078–0.156	0.156–0.313	0.313
	10^3^	0.078	0.156	0.313

**Table 3:** MIC_50_, MIC_90_ and MBC—reported as *v*/*v* percentages—values of hydrogen peroxide against coagulase-negative staphylococci (CoNS; n = 64) at two different inocula (10^5^ and 10^3^ CFUs/mL).

CoNS (n = 64)	Inoculum (CFUs/mL)	MIC_50_ Range (% *v*/*v*)	MIC_90_ Range (% *v*/*v*)	MBC Range (% *v*/*v*)
*S. epidermidis* (n = 22)	10^5^	0.0025–0.156	0.005–0.313	0.078–0.625
	10^3^	0.0025–0.156	0.005–0.156	0.156–0.313
*S. warneri* (n = 14)	10^5^	0.078–0.156	0.156–0.313	0.313–0.625
	10^3^	0.078–0.156	0.156–0.313	0.156–0.625
*S. hominis* (n = 12)	10^5^	0.01–0.313	0.02–0.625	0.156–1.25
	10^3^	0.02–0.078	0.039–0.156	0.078–0.625
*S. capitis* (n = 9)	10^5^	0.078–0.156	0.156–0.313	0.313–0.625
	10^3^	0.078–0.156	0.156–0.313	0.156–2.5
*S. saprophyticus* (n = 3)	10^5^	0.156	0.313	0.625
	10^3^	0.156	0.313	0.313–0.625
*S. pasteuri* (n = 2)	10^5^	0.156	0.313	0.625
	10^3^	0.156	0.313	0.313–0.625
*S. lugdunensis* (n = 2)	10^5^	0.078–0.156	0.156–0.313	0.156–0.313
	10^3^	0.078	0.156	0.156–0.313

**Table 4:** Time–kill assay results of PVI, H_2_O_2_, and their combination—at intermediate and lower concentrations—against *S. epidermidis* at different exposure times. The number of CFU/mL is expressed as mean ± SEM.

Time (Min)	PVI		H_2_O_2_		PVI + H_2_O_2_		Statistical Analysis
	0.08%	0.32%	0.012%	0.5%	0.08%/0.012%	0.32%/0.5%	Unpaired *t*-Test
0.5	153.0 ± 15.74	10.43 ± 6.24	176.4 ± 15.90	85.57 ± 17.79	137.7 ± 16.66	0.86 ± 1.86	0.32% versus 0.08%
1	147.0 ± 11.29	3.43 ± 0.40	155.7 ± 19.32	76.57 ± 16.12	134.0 ± 17.20	0.29 ± 0.76	*p* < 0.001
3	146.3 ± 17.85	2.00 ± 0.11	142.3 ± 9.05	62.67 ± 13.41	128.4 ± 10.10	0.00 ± 0.00	0.5% versus 0.012%
5	121.5 ± 8.63	1.29 ± 0.68	55.33 ± 5.07	40.50 ± 7.21	114.5 ± 10.00	0.00 ± 0.00	ns
10	86.67 ± 5.05	1.00 ± 0.18	39.50 ± 3.89	3.71 ± 8.98	110.9 ± 9.30	0.00 ± 0.00	0.32%/0.5% versus 0.08%/0.012%
							*p* < 0.001

Abbreviations: CFU, colony-forming units; SEM, standard error of the mean; ns, not significant.

**Table 5:** Time–kill assay results of PVI, H_2_O_2_, and their combination—at intermediate and lower concentrations—against *C. acnes* at different exposure times. The number of CFU/mL is expressed as mean ± SEM.

Time (Min)	PVI		H_2_O_2_		PVI + H_2_O_2_		Statistical Analysis
	0.08%	0.32%	0.012%	0.5%	0.08%/0.012%		Unpaired *t*-Test
0.5	107.9 ± 28.11	52.00 ± 15.88	100.5 ± 29.05	16.92 ± 9.91	69.17 ± 18.05	2.42 ± 1.10	0.32% versus 0.08%
1	101.3 ± 26.18	44.80 ± 14.96	99.17 ± 26.02	11.64 ± 7.60	60.60 ± 21.72	0.50 ± 0.36	ns
3	100.4 ± 32.16	43.67 ± 17.18	87.82 ± 19.56	11.82 ± 6.45	57.14 ± 21.63	0.17 ± 0.11	0.5% versus 0.012%
5	90.13 ± 32.88	37.33 ± 15.11	66.50 ± 24.28	4.80 ± 3.88	54.67 ± 18.49	0.17 ± 0.11	*p* < 0.001
10	83.89 ± 21.45	34.33 ± 13.07	58.27 ± 17.75	1.92 ± 1.12	48.11 ± 16.71	0.25 ± 0.25	0.32%/0.5% versus 0.08%/0.012%
							*p* < 0.001

Abbreviations: CFU, colony-forming units; SEM, standard error of the mean; ns, not significant.

**Table 6:** Percentages of mature biofilm disruption of different PVI and H_2_O_2_ combination—at intermediate and higher concentrations—against *S. epidermidis* and *C. acnes* at different exposure times (as minutes).

Bacterium	Time	Low Concentration Combination		High Concentration Combination	
*S. epidermidis*		PVI 0.35% + H_2_O_2_ 0.5%	H_2_O_2_ 0.5% + PVI 0.35%	PVI 5% + H_2_O_2_ 1.5%	H_2_O_2_ 1.5% + PVI 5%
	5′ + 5′	33%	44%	34%	59%
	15′ + 15′	44%	52%	48%	62%
*C. acnes*					
	5′ + 5′	43%	46%	49%	52%
	15′ + 15′	45%	47%	50%	55%

## Data Availability

All the data generated or analyzed during this study are included in this published article.
